# The effect of visfatin on the transcriptomic profile of porcine anterior pituitary cells during periimplantation period

**DOI:** 10.1038/s41598-025-00766-0

**Published:** 2025-05-06

**Authors:** Kamil Dobrzyn, Grzegorz Kopij, Marta Kiezun, Ewa Zaobidna, Marlena Gudelska, Barbara Zarzecka, Katarzyna Kisielewska, Agnieszka Rak, Nina Smolinska, Tadeusz Kaminski

**Affiliations:** 1 Department of Zoology, Faculty of Biology and Biotechnology, University of Warmia and Mazury in Olsztyn, Oczapowskiego st. 5, Olsztyn, 10-719 Poland; 2 Department of Animal Anatomy and Physiology, Faculty of Biology and Biotechnology, University of Warmia and Mazury in Olsztyn, Olsztyn, Poland; 3 Department of Biochemistry, Faculty of Biology and Biotechnology, University of Warmia and Mazury in Olsztyn, Olsztyn, Poland; 4 Department of Human Histology and Embryology, School of Medicine, Collegium Medicum, University of Warmia and Mazury in Olsztyn, Olsztyn, Poland; 5 Laboratory of Physiology and Toxicology of Reproduction, Institute of Zoology and Biomedical Research, Jagiellonian University in Krakow, Krakow, Poland

**Keywords:** Anterior pituitary, Early gestation, Pig, RNAseq, Visfatin, Reproductive biology, Physiology, Gene expression

## Abstract

Females’ reproductive capacity is closely related to the actual metabolic status of the organism. The pituitary, an element of the regulatory hypothalamic-pituitary-ovarian axis, is one of the most important endocrine glands regulating reproductive system activity. Undisturbed functioning of pituitary ensures the regular course of pregnancy through, among others, the modulation of steroid hormones production, which is critical in the early stages of gestation. Visfatin, a hormone belonging to the adipokines family, may belong to a group of factors regulating the reproductive functions in response to the female’s metabolic status. Herein we verified the hypothesis assuming a modulatory effect of visfatin on the porcine anterior pituitary transcriptome on days 15 to 16 of gestation (beginning of implantation). RNA-seq analysis of the porcine anterior pituitary cells revealed changes in the expression of 203 genes (121 up-regulated and 82 down-regulated, when compared to the non-treated controls), assigned to 325 gene ontology terms. The presence of visfatin affected the frequency of alternative splicing events (194 cases), as well as long noncoding RNA expression (64 cases). Visfatin expression and the occurrence of alternative splicing events of genes that are responsible, directly or indirectly, for regulation of the secretory functions of the pituitary, including those critical for reproductive functions suggests that the adipokine may be a key agent in ensuring the appropriate hormonal milieu during the peri-implantation period.

**Supplementary Information:**

The online version contains supplementary material available at 10.1038/s41598-025-00766-0.

## Introduction

Pregnancy is a unique and critical period in a female’s life during which the whole organism is mobilized and focused on ensuring the successful survival and development of the embryo. The main regulatory system responsible for the reproductive functions during both the oestrous cycle and pregnancy is the hypothalamic-pituitary-gonadal (HPG) axis. The axis consists of three branches. The first component, hypothalamus, is responsible for secreting gonadotropin realising hormone (gonadoliberin; GnRH) which stimulates the pituitary cells to secrete gonadotrophins (LH and FSH), whose, carried by the bloodstream, act on the target reproductive tissues. Communication between the branches of the axis occurs through several feedback loops, which ensure homeostasis maintenance^1,2^.

The pituitary gland, a central hub for several regulatory axes originating in the hypothalamus, generally consists of two primary components, the nervous and the glandular part that in pigs constitute separate lobes. This dual structure of the gland enables it to integrate the nervous signals from the CNS and convert them into the hormonal response.

For successful embryo survival during early pregnancy, numerous critical processes must occur in a precise sequence and timing. A successful embryo implantation onto the maternal endometrium can occur only during a specific short period known as the ‘window of implantation’, when uterine tissues are optimally receptive to the embryo^3,4^. The development of uterine receptivity, which involves tissue remodeling and blood vessel development, relies on a hormonal milieu regulated by both local and central mechanisms^5^.

The successful maintenance of pregnancy relies on the efficient integration of the reproductive system and energy metabolism. The correlation between the metabolic health of females and the likelihood of successful delivery, as well as litter size, has been consistently demonstrated. Research indicates that during early gestation, specifically between days 12 to 30 of pregnancy, as much as 30% of porcine embryos may be lost^6^. One potential explanation for this embryo loss could be insufficient energy resources of maternal organism or disruptions in energy metabolism. Hence, it is plausible that embryonic loss during the peri-implantation period could be attributed to a natural mechanism linking litter size with the metabolic status of the female.

The coordination between metabolism and reproductive functions is facilitated by various hormonal factors, with adipokines playing a crucial role. Adipokines are hormones primarily secreted by white adipose tissue. Visfatin (VIS), a protein product of the *NAMPT* gene, exhibits insulin-like effects and belongs to the adipokines family^7^. *NAMPT* encodes a 52–54 kDa protein that can exist in two distinct isoforms within the body: intracellular (iNAMPT) and extracellular (eNAMPT, VIS)^8-10^. Of these forms, only VIS demonstrates hormonal activity, while iNAMPT functions as an intracellular enzyme. To date, no specific receptor for VIS has been identified.

Visfatin has been found to exert pleiotropic actions in the organism^11-17^. The expression of the *NAMPT* gene has been confirmed in reproductive organs such as ovaries and testes, as well as in the pituitary and hypothalamus, suggesting its potential role in regulating reproductive functions^17-20^. Our previous research indicated that VIS has a modulatory effect on the secretory functions of porcine anterior pituitary cells (APc)^21^. These findings prompted us to investigate whether VIS could influence the transcriptomic profile of porcine APc during the mid-luteal phase of the oestrous cycle. Our study concluded that the hormone may impact a broader range of processes beyond reproductive functions^22^. However, there is a lack of research on the potential effects of VIS on gene expression during the peri-implantation period. The research presented here aims to validate the hypothesis assuming that VIS plays a crucial regulatory role in modulating global gene expression in porcine APc, not only during the oestrous cycle but also during the peri-implantation period.

## Materials and methods

### Animals and tissue collection

The studies were conducted in accordance with the Polish Act on the Protection of Animals Used for Scientific or Educational Purposes (15 January 2015, Polish Journal of Laws of 2015, Article 266) and Directive 2010/63/EU of the European Parliament (22 September 2010) concerning the protection of animals used for scientific purposes. The study was designed in accordance with ARRIVE guidelines. All experimental protocols were approved by a named institutional and/or licensing committee. The glandular parts of the pituitary glands (AP) were obtained from gilts (aged 7–8 months and weighting 130–150 kg) on days 15 to 16 of gestation, as they were being prepared for slaughter at the commercial slaughterhouse. The gilts were monitored daily for oestrus behaviour, with the day of onset of the second oestrus designated as day 0 of the oestrous cycle. Mating occurred on days 1 to 2 of the oestrous cycle, with the day following mating marked as the first day of pregnancy. The stage of gestation was confirmed by the presence, size, and morphology of conceptuses obtained through flushing from the uterine horns^23^. Tissue samples were harvested from animals intended for commercial slaughter and meat processing, and the collected tissues were an abattoir by-product. Sows were stunned by electricity (electronarcosis) and bled to death by cutting the carotid artery (exsanguination) in the commercial slaughter, according to European legislation (EFSA, AHAW/04–027). Immediately after slaughter, the AP were transported in ice-cold phosphate buffer solution (PBS) enriched with glucose and a mix of antibiotics (Antibiotic Antimycotic Solution, Merck, USA). Due to the low yield of APc (approximately 9 to 10 million) isolated from a single gland was low, two to three anterior pituitary lobes were collected, digested together, and, after cell counting, the suspension was divided into two parts and seeded on 6-well plates at a concentration of 1.5 × 10^6^ cells/well in 2 mL of medium. Half of the seeded cells were used as a control sample, while the other half was used as a test sample treated with VIS (*n* = 1 per group). In vitro cell cultures were performed in five independent replicates (*n* = 5). Each replicate consisted of cells isolated from different animals and then pooled.

### *In vitro cell cultures and total RNA isolation*

APc were isolated and pre-incubated as previously described^22^. The total number and viability of the APc were determined using a haemocytometer and the Trypan blue dye exclusion method (Sigma-Aldrich, USA). The mean percentage of viable cells after isolation was 94.5% ± 1%. Following the pre-incubation, the culture media were replaced with fresh, serum-free McCoy’s 5 A medium (Sigma-Aldrich, USA), and incubated for 24 h at 37 °C, in a water-saturated atmosphere containing 5% CO_2_ and 95% air. The incubation occurred in the presence of recombinant VIS at a concentration of 100 ng/mL (Cat. # 8424-VF, RD System, USA). The hormone concentration was determined based on our preliminary tests. Cells in the control group were incubated in medium without any treatment. The potential effect of VIS on cell viability was assessed using the alamarBlue™ assay, with the mean percentage of viable cells after incubation being 94% ± 4.3%.

After in vitro culture, total RNA was extracted from the APc using Extrazol (BLIRT, Poland), following the manufacturer’s instructions. The purity (A260/A280) and quantity (A260) of the extracted RNA were determined spectrophotometrically using the Infinite M200 Pro (Tecan, Männedorf, Switzerland). RNA integrity was assessed using a Bioanalyzer 2100 (Agilent Technology, USA). Samples with an RNA integrity number (RIN) greater than 8 were validated for RNA-Seq analysis as well as for PCR and quantitative real-time PCR (qPCR).

### Library construction and sequencing

The procedure for ribosomal RNA (rRNA) removal and library construction was described in our previous study^22^. Libraries were prepared using the TruSeq Stranded mRNA Library Prep Kit (Illumina, San Diego, CA, USA) according to the manufacturer’s protocol. The 2 × 150 paired-end sequencing analysis was performed on the NovaSeq 6000 sequencing system from Illumina (Illumina, San Diego, USA), with a minimum sequencing depth of 40 million reads per sample. The analysis was conducted for porcine APc in five biological replicates for both VIS-treated and control groups (*n* = 5).

### Bioinformatic analysis

To investigate the regulatory mechanisms of porcine APc affected by VIS, we analysed the expression of genes, long noncoding RNAs (lncRNAs) and alternatively spliced (AS) transcripts. The in silico analyses were performed as described in Kopij et al.^24^ and Dobrzyn et al.^22^. Data visualization was conducted using the SRplot online tools (http://www.bioinformatics.com.cn/).

### Transcripts assembly and differentially expressed transcripts processing

Reads containing adapters, and those with low-quality scores (Q_Phred_ score ≤ 20; reads whith more than 5% of unknown nucleotides) were removed using Cutadapt (v.1.9)^25^ and custom Python scripts. The quality of the remaining raw reads was assessed using FastQC (v. 0.11.9)^26^. The HISAT2 (v.2.0.4)^27^ script was employed to map the obtained reads to the *Sus scrofa domestica* genome (v.107) as uploaded in the Ensembl database^28^. Mapped reads were assembled using StringTie (v.1.3.4d)^29^. To reconstruct a comprehensive transcriptome, the transcriptomes from all samples were merged using custom Python scripts and gffcompare (v.0.9.8)^30^. StringTie and Ballgown^31^ were utilised to estimate the expression levels of all transcripts.

Expression levels for mRNAs and lncRNAs were determined by calculating FPKM (fragments per kilobase of transcript per million fragments mapped) with the StringTie script. Differences in the expression of mRNAs (differentially expressed genes; DEGs) and lncRNAs (differentially expressed lncRNA; DELs) between the control and VIS-treated groups were assessed using DESeq2 ^32^. DEGs and DELs with an absolute log_2_ of fold change (log_2_FC) ≥ 0.56 and *p* < 0.05 were considered differentially expressed.

### Functional annotation of target genes

Gene set enrichment was analysed using GSEA software ver. 4.1.0 ^33^ and the Molecular Signatures Database (MSigDB). Enrichment ontology and pathway analysis were performed using the Kyoto Encyclopedia of Genes and Genomes (KEGG)^34^ and Gene Ontology (GO)^35,36^ databases. The gene expression matrix and ranked genes were input using the Signal2Noise normalisation method. KEGG and GO terms with *p* < 0.05 were considered significant.

### Identification of LncRNAs, target gene prediction, and functional analysis

The procedure for identifying lncRNA candidates was conducted as described previously^22,24^ The sequences were analysed using the Coding Potential Calculator (CPC; v. 0.9-r2)^37^ and the Coding-Non-Coding Index (CNCI; v.2.0)^38^ tools to predict transcripts with coding potential. Transcripts with a CPC score < 0.5 and a CNCI score < 0 were classified as novel lncRNAs; the remaining transcripts were categorized as known lncRNAs. To identify potential *cis* interactions of lncRNAs with neighboring genes, a custom Python script selected 100,000 upstream and downstream coding genes. DESeq2 software was used to identify differences in the expression of lncRNA sequences between probes. The functional analysis of target genes for lncRNAs was performed, considering results with a false discovery rate (FDR; q) < 0.05 as significant. The revealed DELs were classified into one of five categories according to StringTie^29^ software class code: (i) intronic, a transfrag falling entirely within a reference intron; (j) potentially novel isoform, or fragment at least one splice junction is shared with a reference transcript; (o) generic exonic overlap with a reference transcript; (u) unknown, intergenic transcript; (x) exonic overlap with a reference on the opposite strand (antisense). Close localisation of DELs and DEGs on the same chromosome (within 100,000 bp) was classified as *cis* interaction. Results with FDR (q) < 0.05 were considered as statistically significant.

### Alternative splicing analysis

To identify AS events and analyze differential alternative splicing events (DASs) between control and VIS-treated groups, we applied the replicate multivariate analysis of transcript splicing (rMATS; v.4.1.1) script^33^.

Trimmed reads with lengths of 120 bp were used to estimate the percent splicing inclusion (PSI) across splicing sites at intron/exon junctions. AS events characterised by q < 0.05 and the absolute value of the inclusion level difference (|ΔPSI|) > 0.1 were considered significant. The identified DASs were classified into one of the following categories: alternative 3′ splice site (A3SS), 5′ splice site (A5SS), mutually exclusive exons (MXE), retention intron (RI), and skipping exon (SE).

### Validation of DEGs and DELs using qPCR

To confirm the results of NGS sequencing, we performed qPCR analysis for selected DEGs and DELs. The analysis was conducted using the Aria Mx Real-time PCR System (Agilent Technology, USA) as described previously^39^. Specific primer pairs and reaction conditions used to amplify parts of the chosen DEGs and DELs, as well as the reference genes ubiquitin C (*UBC*) and 18 S ribosomal RNA (*18sRNA*), are presented in Supplementary Table 1. The reaction mixture contained cDNA, primers, Sensitive RT HS-PCR Mix SYBR (12.5 µL; A&A Biotechnology, Poland), ROX (0.24 µl; reference dye), and RNase-free water achieving a final volume of 20 µL. Non-template controls (NTC) were prepared by substituting cDNA with RNase-free water, or by omitting the reverse transcription step. All reactions were performed in duplicates. The specificity of qPCR reactions was confirmed by melting-curve analysis, and the purity of the products was validated through agarose gel electrophoresis. The results were calculated using the comparative cycle threshold method (ΔΔCT) and normalized with the geometric means of the reference genes’ Ct values^40^. The normality of qPCR data distributions was verified using the Shapiro – Wilk test (*p* > 0.05). The results were analyzed with the Student’s t-test (Statistica software; Statsoft Inc., Tulsa, OK, USA), with values of *p* < 0.05 being considered significant.

### Validation of DAS using PCR

The characterisation of SE events in three selected genes - apoptotic peptidase activating factor 1 (*APAF1*), cyclin D binding myb-like transcription factor 1 (*DMTF1*), and multiple EGF-like domains 10 (*MEGF10*) was performed using StartWarm HS-PCR Mix (A&A Biotechnology, Poland) on a Labcycler 48s (Syngen Biotech, Poland). The reaction mixture (final volume of 25 µL) contained 12.5 µL of Hot Start PCR Mix, pairs of specific primers, nuclease-free deionised water, and cDNA. NTC was prepared as described above. The reaction conditions and primer sequences are detailed in Supplementary Table 1. The resulting PCR products were analysed on agarose gels containing Midori Green Advance dye (Nippon Genetics Europe, Germany).

## Results

### Quality control and statistics of reads

A total of 492,454,032 raw paired-end reads were generated, resulting in an average number of 49,245,403 reads per sample. Alignment to the *Sus scrofa* reference genome showed coverage ranging from 64 to 78.7%. On average, 70.9% of the reads were uniquely mapped, while 1.16% mapped to multiple loci. Of the mapped reads, 55.55% corresponded to coding DNA sequence regions (CDS), while the remainder belonged to introns (40.59%) and intergenic regions (3.68%). Detailed statistics for individual samples can be found in Supplementary Table 2. Raw reads were deposited in the Functional Genomics Data Collection (ArrayExpress) database under the common project accession number E-MTAB-14347.

### DEGs and functional annotations (gene ontology, GO; KEGG)

**Fig. 1:**
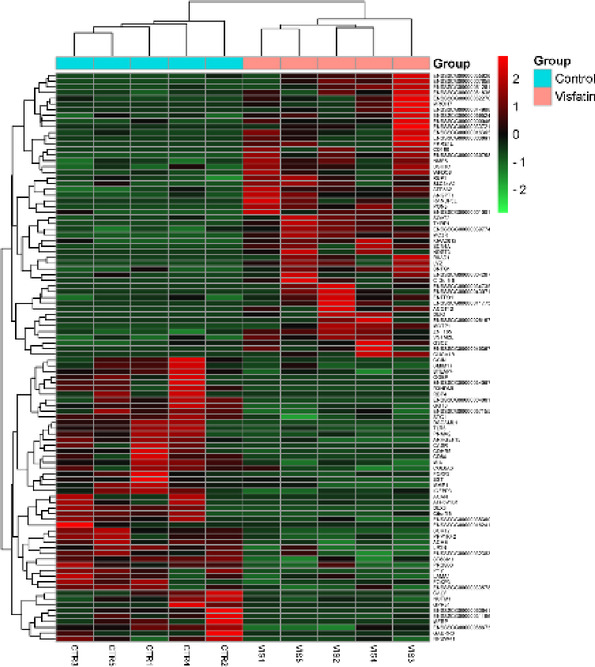
Heatmap of hierarchical clustering for selected genes whose expression was altered by visfatin treatment. Each column represents biological replicates of the control (CTR1-5) or visfatin-treated groups (VIS1-5). The colour brackets correspond to normalised expression values (Z-score; red-green scale) for differentially expressed genes in each biological replicate.

**Fig. 2:**
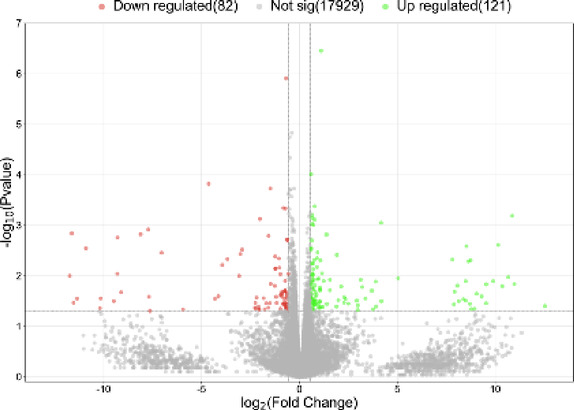
Visualization of the number of genes with altered expression in the presence of visfatin. The X-axis represents logarithmic fold changes in expression (log_2_FC), while the Y-axis shows the negative decimal logarithm of p-values. The horizontal line indicates the negative logarithmic p-value cut-off (*p* = 1.3), and the vertical lines denote the fold change cut-offs (log_2_FC > |0.56|).

**Fig. 3:**
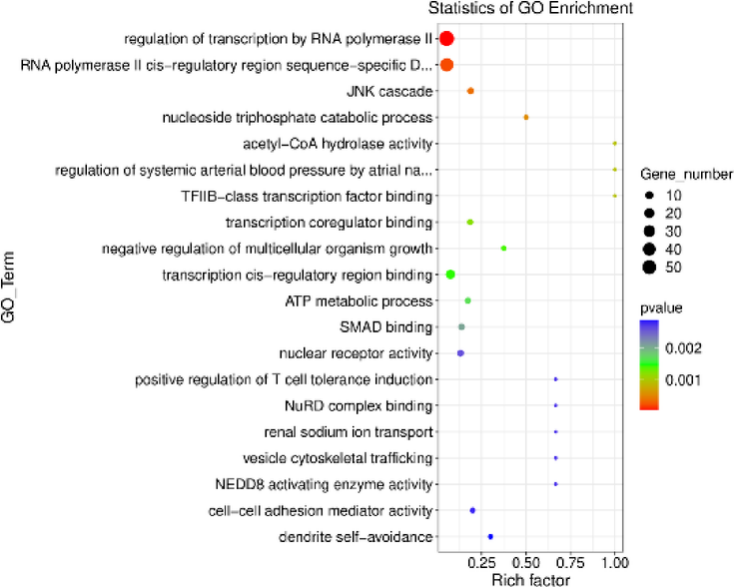
Scatterplot diagram illustrating the enrichment of Gene Ontology (GO) terms with differentially expressed genes (DEGs) influenced by visfatin (*p* < 0.05, log_2_FC>|0.56|). The vertical axis lists the identified GO terms, while the horizontal axis shows the enrichment score, calculated based on the negative decimal logarithm of the term’s p-value. Dot size indicates the number of DEGs involved in each term.

**Fig. 4:**
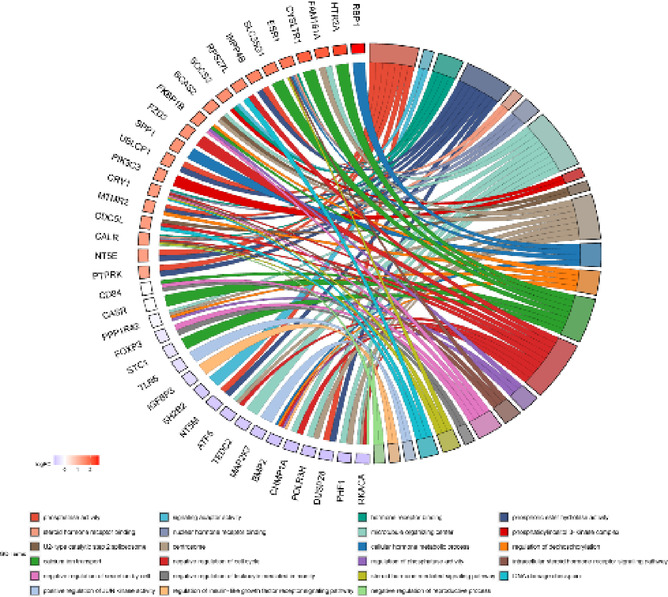
Visualization of significant differentially expressed genes (DEGs) and their enrichment in ontology terms. The circos plot displays selected Gene Ontology (GO) terms associated with DEGs in porcine anterior pituitary cells, whose expression was altered by visfatin (*p* < 0.05, log_2_FC>|0.56|). The colours of the lines represent different GO terms, while the bars corresponding to gene symbols indicate the ratio of upregulated to downregulated genes. Red bars indicate GO terms with predominantly upregulated DEGs, while blue bars indicate those with predominantly downregulated DEGs.

Among the 18,132 genes identified, 203 met the criteria of log_2_FC > |0.56| and *p* < 0.05. The results are illustrated in Figs. 1 and 2. Among the identified DEGs, 121 showed increased expression, while 82 exhibited decreased expression in the presence of VIS. The log_2_FC values ranged from − 11.74 (*ENSSSCG00000060641*) to 12.54 (*ENSSSCG00000026167*) (Supplementary Table 3). The identified DEGs were classified into three GO categories: ‘biological processes’ (BP), ‘cellular components’ (CC), and ‘molecular function’ (MF). In total, DEGs were associated with 325 GO terms (p < 0.05), with 198 terms linked to BP, 30 to CC and 92 to MF categories. Five of the identified terms were not assigned to any of the aforementioned categories (Supplementary Table 4). The majority of DEGs in the BP category were associated with *regulation of transcription by RNA polymerase II* (GO:0006357; 57 DEGs) and *regulation of DNA-templated transcription* (GO:0006355; 46 DEGs). In the CC category, the most enriched terms included *extracellular space* (GO:0005615; 38 DEGs) and *extracellular matrix* (GO:0031012; 13 DEGs). For the MF category, the largest number of DEGs was associated with *identical protein binding* (GO:0042802; 48 DEGs) and *RNA polymerase II cis-regulatory region sequence-specific DNA binding* (GO:0000978; 43 DEGs). The results of the GO analysis are presented in Fig. 3, while Fig. 4 highlights the enrichment of GO terms along with selected key DEGs.

**Fig. 5:**
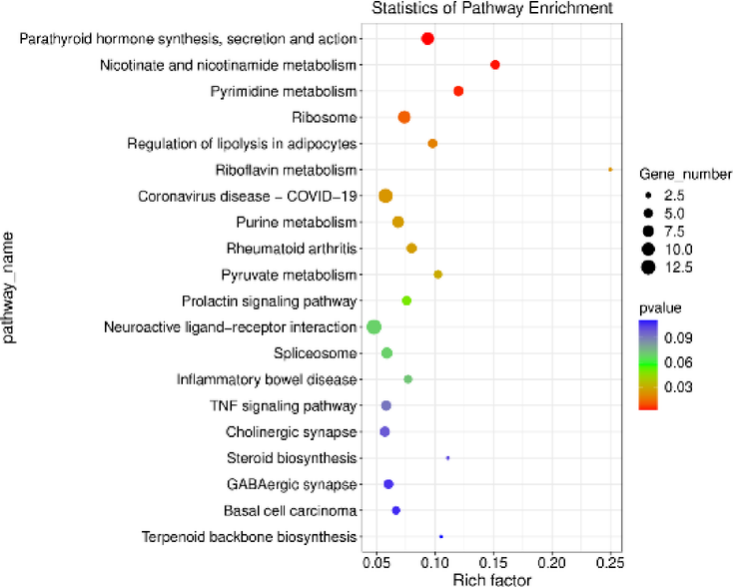
Scatterplot diagram illustrating the enrichment of Kyoto Encyclopedia of Genes and Genomes (KEGG) pathways with differentially expressed genes (DEGs) influenced by visfatin (*p* < 0.05, log_2_FC>|0.56|). The vertical axis lists the identified pathways, while the horizontal axis shows, the enrichment score, calculated based on the negative decimal logarithm of the pathway’s p-value. Dot size indicates the number of DEGs involved in each pathway.

KEGG database analysis identified 11 signalling pathways (*p* < 0.05), with the most enriched pathways being *Neuroactive ligand-receptor interaction* (ko04080; 13 DEGs) and *Coronavirus disease - COVID-19* (ko05171; 12 DEGs). The results of the KEGG analysis are displayed in Fig. 5. Detailed findings form the analysis are provided in Supplementary Table 4.

### Identification of LncRNAs, target gene prediction and functional analysis

**Fig. 6:**
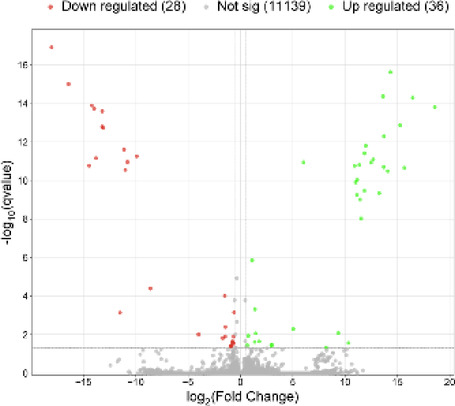
Visualization of the number of lncRNNA with altered expression in the presence of visfatin. The X-axis represents logarithmic fold changes in expression (log_2_FC), while the Y-axis shows the negative decimal logarithm of q-values. The horizontal line indicates the negative logarithmic q-value cut-off (q = 1.3), and the vertical lines denote the fold change cut-offs (log_2_FC > |0.56|).

From a total of 11,204 identified lncRNAs candidates, 64 met the criteria of FDR < 0.05 and log_2_FC > |0.56|, classifying them as DELs. Among these, 38 were annotated with ENSEMBL gene IDs, while the remaining 26 were identified as novel lncRNAs. In the group of DELs, 36 were found to be upregulated, and 28 were downregulated in the VIS-treated group (Fig. 6. and Supplementary Table 5). The interactions analysis revealed a total of 69 *cis* interactions. Of these, 54 indicated co-localization within a range of 100,000 bp, 13 were in close proximity (10,000 bp), and 2 were in very close proximity (1,000 bp). Additionally, four of the identified lncRNAs exhibited a strong correlation with their target genes (Pearson Correlation Coefficient > 0.7), however these target genes did not meet the criteria to be classified as DEGs. Therefore, no significant DEL–DEG pairs were identified. As a result, data concerning the co-localization of protein-coding genes and DELs were excluded from further functional analyses, and the GO analysis focused on genes associated with DELs that did not qualify as DEGs.

**Fig. 7:**
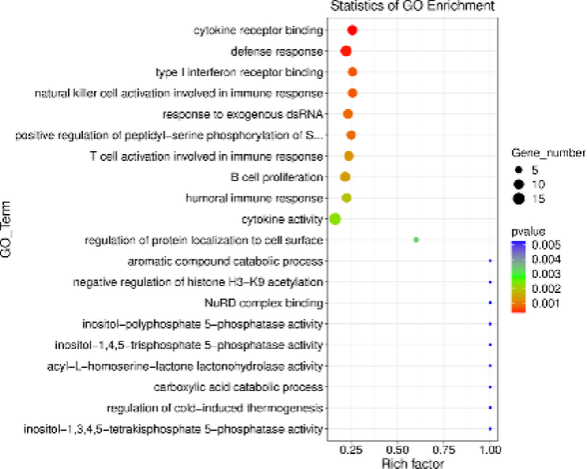
Scatterplot diagram illustrating the enrichment of Gene Ontology (GO) terms with differentially expressed long noncoding RNA’s (DELs) influenced by visfatin (*p* < 0.05, log_2_FC>|0.56|). The vertical axis lists the identified GO terms, while the horizontal axis shows the enrichment score, calculated based on the negative decimal logarithm of the term’s p-value. Dot size indicates the number of DELs involved in each term.

The GO analysis revealed 67 processes involving genes linked to the identified lncRNAs. Among these, 43 processes were associated with the BP category, 9 with the CC category, and 13 with the MF category. Within the BP category, the most enriched processes included *defense response to virus* (GO:0051607; 13 DELs) and *defense response* (GO:0006952; 12 DELs). In the CC category, the most enriched terms were *growth cone* (GO:0030426; 5 DELs), *keratin filament* (GO:0045095; 4 DELs), and *melanosome* (GO:0042470; 4 DELs). Lastly, the MF category highlighted *cytokine activity* (GO:0005125; 15 DELs) and *cytokine receptor binding* (GO:0005126; 10 DELs). The results of the GO analysis for DELs are illustrated in Fig. 7 and detailed in Supplementary Table 6.

### Alternative splicing analysis results

**Fig. 8:**
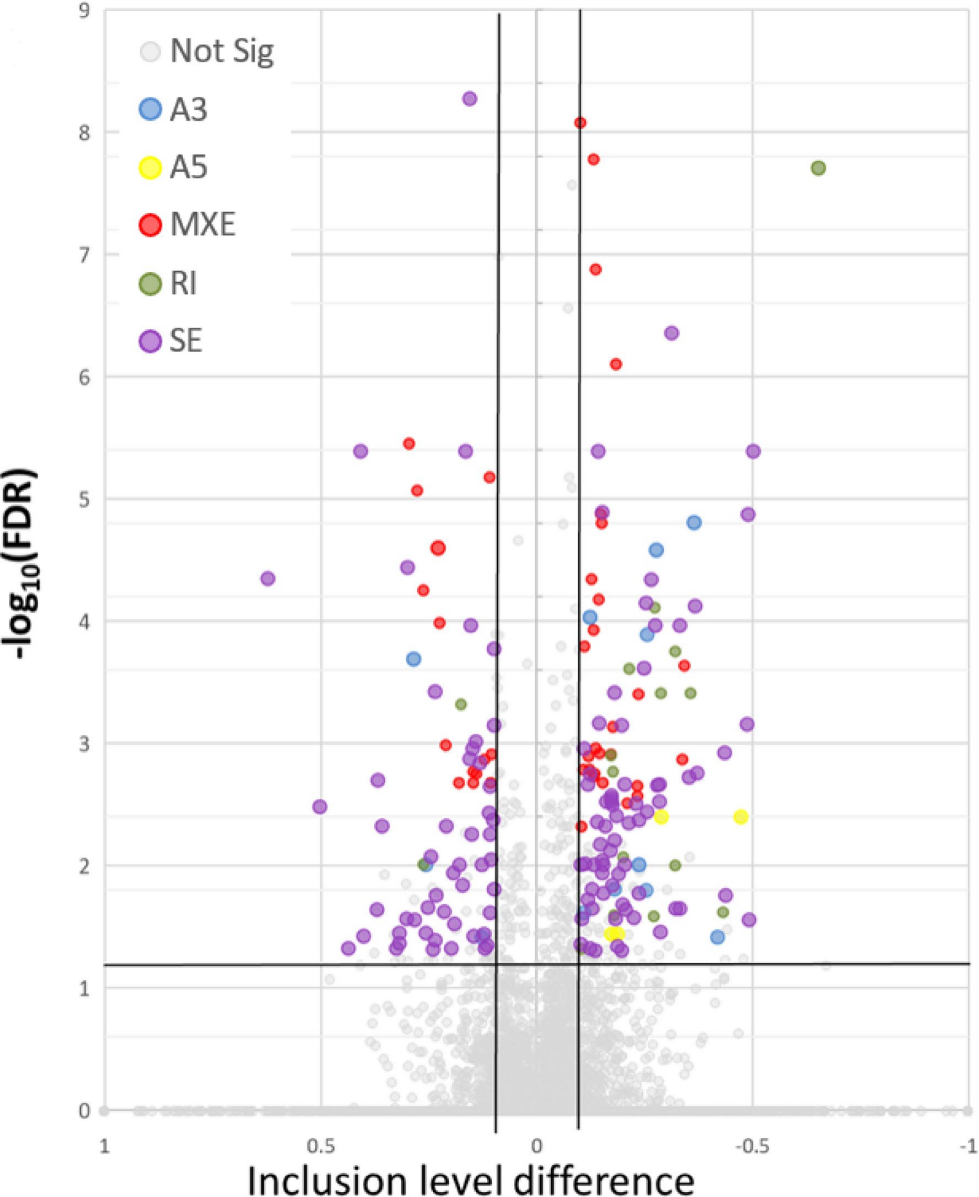
Visualization of alternative splicing (AS) events in the transcriptome of the anterior pituitary cells (APc) under the influence of visfatin. (A) Volcano plot showing the differences in AS events frequency between control and visfatin-treated groups. The X-axis represents ΔPSI-values for each AS event, and the Y-axis shows the negative decimal logarithm of the adjusted p-value. The horizontal line indicates the negative logarithmic adjusted p-value cut-off (q = 1.3), while the vertical lines represent the ΔPSI-value cut-off (ΔPSI>|0.1|). Different colours of dots represent different types of AS events: blue — alternative 3′ splicing sites (A3SS), yellow — alternative 5′ splicing sites (A5SS), red — mutually exclusive exons (MXE), dark green — retention intron (RI), and purple — skipping exon (SE), grey – not significant (Not Sig).

The analysis of AS occurrence identified a total of 19,865 events, of which 194 were classified as DAS with |ΔPSI|>0.1 (Fig. 8). Among these events, we detected 12 A3SS, 4 A5SS, 43 MXE, 16 RI, and 119 SE events. Detailed results of the DASs analysis can be found in Supplementary Table 7.

### qPCR and PCR analyses results

**Fig. 9:**
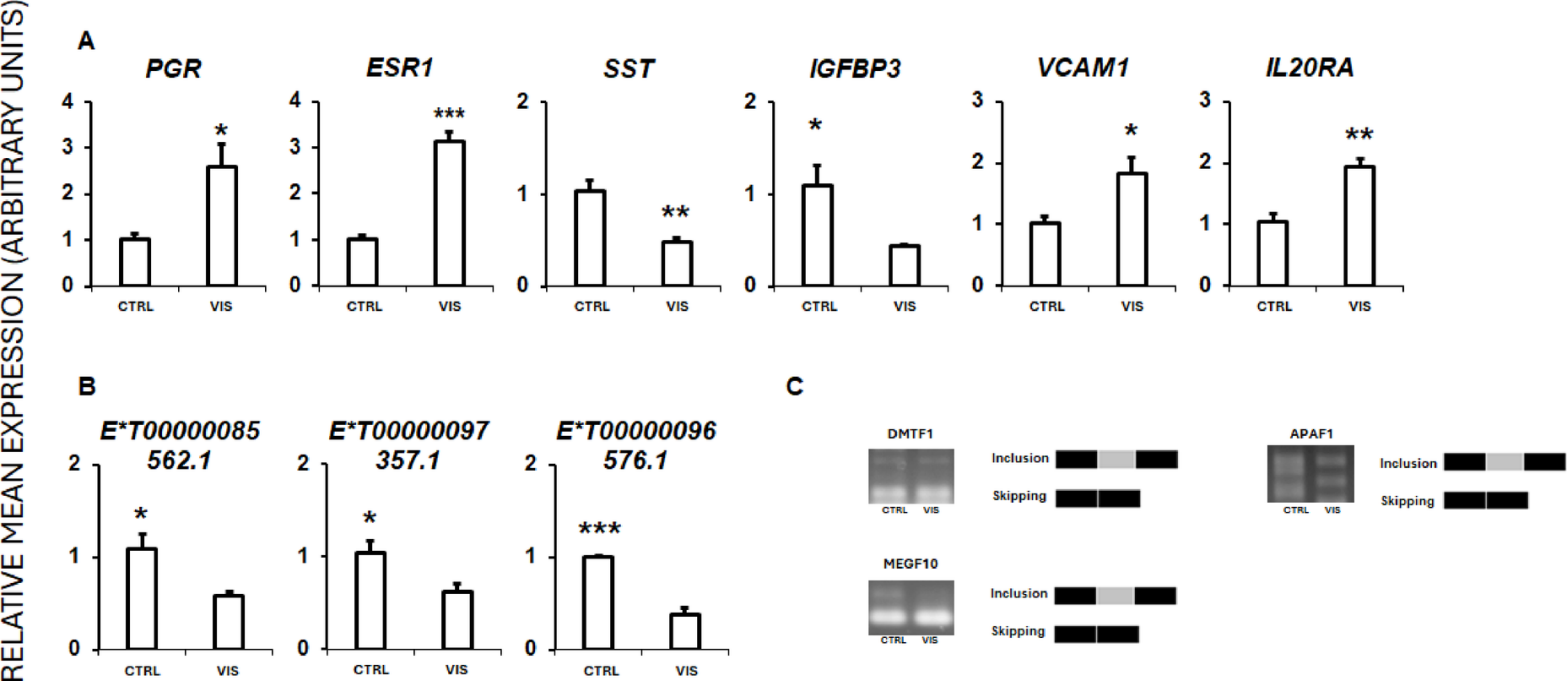
Quantitative real-time PCR (qPCR) and PCR validation of RNA-seq results for differentially expressed genes (DEGs) (A), long noncoding RNAs (DELs) (B), and alternative splicing events (DASs) (C) in visfatin-treated porcine anterior pituitary cells (*n* = 5). (A): Validation of DEGs for PGR, ESR1, SST, IGFBP3, VCAM1, and IL20RA genes. (B): Validation of DELs for ENSSSCT00000085562.1, ENSSSCT00000097357.1 and ENSSSCT00000096576.1 lncRNAs. qPCR validation analyses were performed using UBC and 18sRNA reference genes (* p-value < 0.05; ** p-value < 0.01). (C): PCR validation results of selected DASs. Images show the inclusion and skipping exon levels between the visfatin-treated and control group for DMTF1, APAF1 and MEGF10. Original, uncropped electrophoresis gels are presented in the Supplementary File 1. Abbreviations: CTRL - control group, VIS - visfatin-treated group, PGR - progesterone receptor, ESR1 - oestrogen receptor 1, SST - somatostatin, IGFBP3 - insulin-like growth factor binding protein 3, VCAM1 - vascular cell adhesion molecule 1, IL20RA - interleukin 20 receptor subunit alpha, UBC - ubiquitin C, 18sRNA − 18 S ribosomal RNA, APAF1 - apoptotic peptidase activating factor 1, DMTF1 - cyclin D binding myb-like transcription factor 1, MEGF10 - multiple EGF-like domains 10.

To validate the RNA-seq results, six DEGs were selected for qPCR: progesterone receptor (*PGR*), estrogen receptor 1 (*ESR1*), somatostatin (*SST*), insulin like growth factor binding protein 3 (*IGFBP3*), vascular cell adhesion molecule 1 (*VCAM1*), and interleukin 20 receptor subunit alpha (*IL20RA*). Additionally, three DELs (*ENSSSCT00000085562.1*,* ENSSSCT00000097357.1*, and *ENSSSCT00000096576.1*) were included in the qPCR experiments. For validating the DAS analysis, three AS genes were selected for testing: *DMTF1*, *APAF1*, and *MEGF10*. The qPCR results for both DEGs and DELs (Fig. 9A, B), and the PCR results for DASs (Fig. 9C) confirmed the data obtained from the NGS experiments. These validation results substantiate the accuracy and reliability of the RNA-Seq findings, as well as the methods employed in the data analysis for this study.

## Discussion

To the best of our knowledge, this study represents the first comprehensive examination of the impact of VIS during the peri-implantation period on the APc and is one of the few studies focusing on the analysis of the entire pituitary transcriptome during early pregnancy. The authors acknowledge that, due to the in vitro cell culture model used, the obtained results may not fully reflect the physiological conditions. Although in vitro cultured APc’s lack the hormonal background of a living organism, this model appears to be the most reasonable approach for basic physiological studies in large animals such as the domestic pig. Furthermore, in vitro studies allows for the exclusion of environmental factors, which, in the case of pigs, cannot be controlled as strictly as in small laboratory animals and could otherwise bias the results.

The obtained results revealed the stimulatory effect of VIS on the expression of genes related to the fundamental functions of the pituitary gland, particularly those involved in regulating reproductive functions, such as the *PGR* and *ESR1*, which are part of the ‘nuclear receptor activity’ GO term (GO:0004879). Continuous synthesis of progesterone (P_4_) is essential for establishing and maintaining pregnancy by promoting uterine receptivity development during the ’window of implantation’ and stimulating histotroph secretion^41^. Additionally, oestradiol (E_2_) in pigs serves as a signal for maternal pregnancy recognition and has luteoprotective effects by, *inter alia*, reducing prostaglandin F_2α_ release from uterine tissues into the peripheral circulation^42^. Both hormones regulate reproductive functions not only directly in reproductive tissues but also through a regulatory feedback loop by binding to nuclear receptors in higher branches of the hypothalamic-pituitary-gonadal axis. The increased expression of *PGR* and *ESR1* genes, in conjunction with the known effects of P_4_ and E_2_ on LH and FSH production, suggests the potential role of VIS in modulating the pituitary gland’s sensitivity to steroid hormones and consequently influencing their production in the ovaries^43-45^. The role of VIS in regulating reproductive functions is further supported by its effect on the expression of somatostatin (*SST*) gene. Somatostatin has been shown to reduce the volume and density of gonadotroph-immunoreactive cells in the rats’ pituitary, as well as serum LH and FSH concentrations^46-48^. These findings suggest that VIS may play a role in regulating the reproductive-related functions of the AP by modulating the sensitivity of APc to steroid hormones and regulating gonadotrophin production and secretion. We observed similar results during our studies on the VIS influence on APc during the mid-luteal phase of the oestrous cycle. We noted that VIS also stimulates the expression of *PGR* and modulates the expression of other genes connected with the regulation of AP secretory functions, such as early growth response 2, inhibin E, and PROP Paired-Like Homeobox 1^23^. The altered expression of *ESR1* observed during the implantation period but not in the mid-luteal phase of the oestrous cycle, suggest that VIS may directly regulate the expression of this gene in the context of implantation. Further confirmation can be supported by the inhibitory effect of VIS on the expression of *MSTRG.15015.1* lncRNA, associated with the GRB2-associated binding protein 2 (*GAB2*) gene, which is involved in the regulation of the FSH signalling pathway in granulosa cells^49^.

Additionally, VIS may indirectly influence gonadorophins secretion by modulating the expression of genes involved in extracellular matrix (ECM) composition and cell-to-cell connections. ECM components, including proteins like collagens, laminins, and integrins, provide structural support and communication channels for pituitary cells^50^. Our study revealed that VIS affected a group of genes related to cell adhesion and cell-to-cell contact grouped in ‘extracellular matrix’ (GO:0031012), as well as with ‘cell junction’ (GO:0030054) GO terms. VIS decreased the expression of genes like collagen type VI alpha 3 chain (*COL6A3*) and laminin subunit alpha 1 (*LAMA1*), grouped in the ‘extracellular matrix’ term. Collagen type VI, an essential component of ECM, consists of different alpha chains that play a critical role in regulating cell proliferation, apoptosis, and angiogenesis through the formation of a cell microfilament network and interaction with other ECM molecules^51,52^. These characteristics highlight collagen IV as a significant factor in tumor formation^51,53^, although contradictory effects have also been observed^54^. The overexpression of the *COL6A3* gene in pituitary adenomas has been correlated with the suppression of tumor growth and metastasis capacity^54^. The second of the mentioned genes, *LAMA1*, encodes alpha chain of laminin, a fundamental component of the basement membrane^55^. The presence of laminin as an ECM component has been shown to enhance the formation of gap junctions between pituitary folliculo-stellate cells in vitro^56^. Furthermore, laminin has been demonstrated to impact the secretion of prolactin (PRL) and gonadotrophins from rat APc, indicating its regulatory role in the hormone release.

The modulatory effect of VIS on ECM composition and cell junctions is also evident in the analysis of the alternative splicing in the catenin delta 1 (*CTNND1*) gene, which similarly to other catenins, forms the cadherin–catenin complex responsible for cell-cell adhesion at adherens junctions and participates in the catenin signalling pathway^57^. These seems to confirm VIS involvement in modulating ECM composition, and, as a consequence, in the indirect modulation of pituitary secretory functions.

We also revealed that VIS affects the expression of neuropeptides B and W receptor 1 (*NPBWR1*), peptide YY (*PYY*), and opioid receptor delta 1 (*OPRD1*) genes. NPBWR1, along with NPBWR2, functions as a receptor for neuropeptides B (NPB) and W (NPW)^58,59^, are involved in regulating reproductive processes and pituitary secretory functions^60-62^. This involvement may also be supported by a study on pigs, where NPB was found to modulate the secretion of steroid hormones in porcine Leydig cells and ovarian granulosa cells^63^. NPB has been shown to induce the release of LH in both male and female rats, while NPW has been found to affect the release of PRL, corticosterone, and GH into rat plasma^64,65^. PYY, has also been shown to stimulate gonadotrophins release in rats^66^. Analysis of DEGs revealed altered expression of *OPRD1*, which encodes one of the receptors for endogenous opioids. These opioids not only inhibit GnRH release from the hypothalamus but may also directly influence LH secretion from the APc^67^. Furthermore, studies on opioid receptors in the porcine APc have demonstrated the modulatory role of endogenous opioids in regulating LH and FSH secretion, mediated through the OPRD1 receptor^68^. Moreover, the upregulation of the expression of 5-hydroxytryptamine (serotonin; 5-HT) receptor 2 A (*HTR2A*) gene, was observed in response to VIS. *HTR2A* encodes the serotonin receptor, which has been shown to regulate the production and secretion of gonadotrophins and other pituitary-derived hormones^69-72^. These findings indicate that, through the regulation of the neurotransmitter receptor expression, VIS may modulate the action of 5-HT in the pituitary, subsequently influencing the production of trophic hormones derived from the pituitary. Additionally, the analysis of AS revealed an altered frequency of MXE in the *NPR3* gene, which encodes natriuretic peptide receptor 3 responsible for binding natriuretic peptides and clearing them from circulation^73^. Since natriuretic peptides, which are produced in significant amounts by gonadotrophs, may influence the secretion of pituitary tropic hormones, it is plausible that VIS could modulate the availability of these peptides by altering the expression of specific splicing variants of NPR3. Consequently, this may modulate their overall effect^73-80^. Overall, the modulatory effect of VIS on the expression of receptors for hypothalamic-derived regulatory factors in the APc indicates that this adipokine regulates the secretory function of the pituitary by influencing cellular sensitivity to signals from the hypothalamus.

The other genes influenced by VIS are associated with the regulation of cytokine and growth factor actions. Analysis of DEGs demonstrated that VIS increased the expression of suppressor of cytokine signalling 2 (*SOCS2*), while decreasing insulin-like growth factor binding protein 3 (*IGFBP3*). SOCS2 is part of a family of negative regulatory SOCS proteins that are expressed in response to the activation of cytokine and growth factor signalling cascades primarily through the modulation of Janus kinase-signal transducer and activator of transcription (JAK-STAT) pathways^81^. Within the pituitary gland, SOCS2 has been shown to participate in the regulation of signalling pathways induced by GH and PRL, indicating its role in the secretory functions of the pituitary^82,83^. Moreover, SOCS2 has been reported to interact with the insulin-growth factor-1 (IGF-1) receptor, suggesting its potential modulatory influence on the IGF-1 signalling pathway^84^. IGF-1 is part of a group of peptide growth factors possessing mitogenic, anti-apoptotic, and differentiating properties, which, alongside GH, forms the GH/IGF axis. This axis operates through a regulatory feedback loop that inhibits GH production^85^. Meanwhile, IGFBP3 binds IGF-1, regulating its availability and subsequent actions in target cells^86^. The modulatory effect of VIS on the expression of both *SOCS2* and *IGFBP3* supports the hypothesis of its potential role as a regulator of secretory functions in the APc. Additionally, data concerning the influence of adipokine on the occurrence of RI type AS in the *STAT1* gene further corroborates this assertion.

## Conclusions

The results presented here may indicate, for the first time, the regulatory effect of VIS on the porcine APc during early gestation. VIS influences a substantial number of genes and the occurrence of AS events in various genes that are responsible, either directly or indirectly, for regulating the secretory functions of the pituitary, including those critical for reproductive functions such as LH, FSH, and PRL. Considering that the maintenance of pregnancy relies on the continuous production of P_4_, tightly regulated by the HPG axis, we conclude that VIS is a key agent in ensuring the appropriate hormonal milieu during the peri-implantation period. However, to fully confirm the above conclusions, the obtained results should be corroborated by further studies focusing on the mechanisms through which VIS affects the pituitary gland, as well as determining whether the results reflect changes in the proteomic and metabolomic profiles of APc. A crucial challenge for fully understanding the mechanism of VIS action will be the final identification of its still unknown receptor.

## Electronic supplementary material

Below is the link to the electronic supplementary material.

Supplementary Material 1

Supplementary Material 2

Supplementary Material 3

Supplementary Material 4

Supplementary Material 5

Supplementary Material 6

Supplementary Material 7

## Data Availability

The datasets supporting the conclusions of this article are available in the Functional Genomics Data Collection (ArrayExpress) repository and can be accessed with the E-MTAB-14347 accession number.
